# Neurodisability among Children at the Nexus of the Child Welfare and Youth Justice System

**DOI:** 10.1007/s10964-020-01234-w

**Published:** 2020-04-16

**Authors:** Susan Baidawi, Alex R. Piquero

**Affiliations:** 1 Department of Social Work, Faculty of Medicine, Nursing & Health Sciences, Monash University, PO Box 197, Caulfield East, VIC 3145 Australia; 2 Program in Criminology & Criminal Justice, The University of Texas at Dallas, 800 W. Campbell Rd., GR31, Richardson, TX 75080-3021 United States; 3 Criminology, School of Social Sciences, Monash University, Clayton Campus, VIC 3800 Australia

**Keywords:** Crossover youth, Neurodisability, Child welfare, Juvenile justice, Delinquency, Residential care

## Abstract

Although neurodisability features significantly across child welfare and youth justice cohorts, little research investigates neurodisability among crossover children with dual systems involvement. This study examined differences in childhood adversity, child protection involvement, and offending among crossover children by neurodisability status. Data were from a sample of 300 children (68% male, 31% female, 1% transgender; mean age = 16.2 years, range 10–21) who were charged and appeared in three Australian children’s courts, and who also had statutory child protection involvement in the study jurisdiction. The results indicated that nearly one-half of crossover children had a neurodisability (48%) and this group experienced greater cumulative maltreatment and adversity, earlier out-of-home care entry and offending onset, more caregiver relinquishment and residential care placement, and a greater volume of charges. While substantial differences between specific neurodisabilities were evident, crossover children with any neurodisability had greater odds of having charges related to criminal damage and motor vehicle theft, however they were no more likely to have violent charges relative to other crossover children. The study’s findings demonstrated that the prevalence of neurodisability, and child welfare system responses to this phenomenon, contributes to several offending-related trends observed among crossover children.

## Introduction

The over-representation of youth from child protection backgrounds in the youth justice system presents long-standing concerns across several jurisdictions internationally, including the US (Jonson-Reid et al. 2018), UK (Shaw 2016), Ireland (Carr and Maycock 2019), Canada (Brownell et al. 2018), Australia (Baidawi and Sheehan 2019a), and New Zealand (Stanley 2017). Children who traverse both child welfare and youth justice systems are alternately referred to in the literature as “crossover”, “dual jurisdiction” or “dually-adjudicated” youth (Baidawi and Sheehan 2019b). This traversal encompasses the greater likelihood of maltreated and child protection-involved children coming into contact with the youth justice system in a broad sense (e.g., being charged by police or convicted by courts), as well as their higher propensity of coming under statutory youth justice system supervision in both community and custodial settings. Across Australia, for instance, the location that forms the basis of the current study, contemporary data indicate that children receiving statutory child protection services due to maltreatment or parental incapacity are nine times more likely to offend and come under the supervision of youth justice services compared to other children in the community (Australian Institute of Health and Welfare 2018). The current study specifically focuses on children with statutory child protection involvement who also come before children’s criminal courts charged with offending.

Aside from their over-representation in the youth justice system, the available data indicate that crossover children experience earlier onset of youth justice system contact, greater likelihood of violent offending, greater continuation of offending into mid-adulthood, and higher recidivism compared to other justice-involved children (Malvaso et al. 2018b; Baglivio et al. 2016; Widom et al. 2018). At the same time, much less work has explored neurodisability among children with dual systems involvement. Accordingly, this study uses a unique sample from Australia in order to examine differences in childhood adversity, child protection involvement, and offending among “crossover” children by neurodisability status.

### Factors Influencing the Maltreatment–Offending Relationship

Evidence to date indicates that crossover children experience relatively serious youth justice involvement. As a group, they display a younger age of offending onset, more violent offending, and are at greater risk of experiencing custodial sentences compared to other justice-involved children (Lee and Villagrana 2015; Malvaso et al. 2019; Baidawi and Sheehan 2019a). While these outcomes are problematic, only a minority of children with protective services contact (between 1 and 10%) have later youth criminal convictions (Malvaso et al. 2017b; Vidal et al. 2017). As such, much research has focused on identifying individual, familial, environmental, and systemic factors increasing the likelihood of *any* youth justice involvement among child protection-involved youth. Sociodemographic predictors of youth justice involvement among maltreated children include male gender and racial minority status (Vidal et al. 2017; Cho et al. 2019; Malvaso et al. 2017b). While any maltreatment experiences increase the likelihood of youth justice contact (Ryan and Testa 2005; Chiu et al. 2011), at greatest risk are children exposed to neglect and physical abuse in particular, as well as those experiencing maltreatment recurrence, or persistence into adolescence (Hurren et al. 2017; Malvaso et al. 2017b; Vidal et al. 2017).

Care system-related risks for justice involvement include any placement in out-of-home care (Malvaso et al. 2017b), and specific placement in residential care or “group home” settings (Cutuli et al. 2016; Ryan et al. 2008). Given that children entering residential care typically experience complex challenges prior to residing in these settings, ongoing debate exists as to the relative impact of past adversity (e.g., childhood maltreatment), and placement-related factors (e.g., staffing) in explaining these outcomes (Cutuli et al. 2016; Shaw 2014). Care placement instability, entry into care for behavioral reasons (as opposed to maltreatment), and older age at care entry each increase the risk of youth justice contact (Baskin and Sommers 2011; Goodkind et al. 2013; Ryan 2012). One process seen to inflate the justice involvement of children in residential care is the criminalization of their challenging behaviors (Gerard et al. 2019; Baidawi and Sheehan 2019c). Such “care-criminalization” (McFarlane 2018) is observed to result in residential care-placed children facing police involvement following relatively minor incidents, such as smashing a mug, unlikely to incur legal sanctions in a family home. Additionally, while quantitative analyses are still emerging, a key concern is that out-of-home care systems may contribute to crossover children’s exposure to offending peers through co-placement in residential care (Shaw 2014).

While a growing body of research has examined risk factors for any justice involvement among maltreated children, fewer studies have investigated variability in these outcomes, particularly identifying the factors related to more serious offending, among crossover children. Identification of these factors is potentially very helpful in assisting child welfare and youth justice policy-makers to develop evidence-informed approaches for responding to children at the interface of child welfare and youth justice systems.

### Neurodisability and Crossover Children

Neurodisability (sometimes termed neurodevelopmental disability/disorder) is an umbrella term for conditions with onset in childhood and adolescence that involve a compromise to the nervous system due to genetic, pre-birth or birth trauma, injury, or illness in childhood (Hughes et al. 2012; American Psychiatric Association 2013b). Such disorders include intellectual disability (ID), other specific learning disabilities (e.g., dyslexia), communication disorders (e.g., language and speech disorders), attention deficit hyperactivity disorder (ADHD), autism spectrum disorder, and fetal alcohol spectrum disorder. Neurodisability often generates complexities in personal, social, academic or occupational functioning due to cognitive delay, communication difficulties, and challenges in emotional and behavioral regulation (Chitsabesan and Hughes 2016; American Psychiatric Association 2013b). This “complex mix of influences”, as Chitsabesan and Hughes (2016), p. 114) argue, contributes to the etiology of neurodisability, including genetics, fetal alcohol exposure, perinatal and postnatal birth trauma or injuries, other childhood trauma, socio-emotional deprivation, nutritional deficiencies, and toxin exposure (e.g., lead) (American Psychiatric Association 2013b). Many of these adverse outcomes have been linked to extreme styles of offending, such as the etiology articulated for life-course-persistent offenders within Moffitt’s (1993) developmental taxonomy.

The phenomenon of neurodisability has received very little attention in research concerning crossover children (Baidawi and Sheehan 2019c). This is a surprising and unfortunate oversight in light of known associations between neurodevelopmental impairments and each of childhood maltreatment, childhood adversity, child protection contact, antisocial behavior, and youth justice involvement (Jones et al. 2012; Stalker and McArthur 2012; Algood et al. 2011; Barrett et al. 2014). Research examining maltreatment and protective services involvement among children with disabilities is hampered by definitional variations concerning both disability and maltreatment (Jones et al. 2012; Sullivan and Knutson 2000), and studies that specifically report on maltreatment among children with neurodisability are negligible. Still, the available evidence indicates that children with certain neurodisabilities (including but not limited to behavioral disorders including autism spectrum disorder and attention deficit hyperactivity disorder, as well as intellectual disability and conduct disorder) are at increased risk of childhood maltreatment and protective services involvement (Maclean et al. 2017; Lange et al. 2013; Hibbard and Desch 2007; Sullivan and Knutson 1998; Clayton et al. 2018; Burke et al. 2011; Jones et al. 2012). For example, a population-based study by Sullivan and Knutson (2000) found a 31% prevalence rate of maltreatment among children with disabilities (compared to 9% for non-disabled children), with the highest prevalence rates reported for children with behavior disorders, speech/language disorders, and intellectual disability. Studies have also identified gender differences in this area, with males being more prevalent among maltreatment victims with disabilities compared to females (Sullivan and Knutson 2000; Hershkowitz et al. 2007).

Alongside from their increased risk of maltreatment, children with neurodisability are substantially over-represented in community and custodial youth justice populations (Hughes and O’Byrne 2016; Snow and Powell 2008). For instance, a US national survey found that 33% of incarcerated children had a disability, with nearly one-half of these noted disabilities relating to neurodisability (e.g., specific learning disabilities and intellectual disability) (Quinn et al. 2005). Of course, correlations between neurodisability and offending should not be taken to imply causation. Specific neuropsychological risks most strongly associated with youth justice involvement appear to be related to challenges in verbal and executive functioning (Moffitt 1993), which are variable even among children with common neurodisability diagnoses. For instance, Moffitt and Henry (1989) found that only a subset of children with attention deficit disorder also experienced executive function deficits. Moffitt (1990) further identified in a longitudinal analysis that compared to boys with attention deficit disorder who did not offend, boys with attention deficit disorder who did offend had experienced greater family adversity. Such findings demonstrate variability between children with neurodisability, and that neurodisability alone does not lead to a greater propensity for offending.

### The Link to Developmental/Life-Course Theory

Several developmental/life-course theoretical frameworks outline the emergence of antisocial behaviors via the interaction between individual neuropsychological factors (e.g., temperament and self-regulation) and adverse rearing environments (e.g., punitive or neglectful parenting) (Moffitt 1993). As described by Moffitt (1993), by virtue of their needs, children with challenges in relation to aggression, attention and emotional regulation, may be more challenging to care for, generating to more negative responses from caregivers and other key figures such as teachers, further denying them the relational environments that could best address their needs. These characteristics and “failed” parent-child encounters are associated with children’s enhanced risk of victimization through abuse or neglect from exasperated caregivers, particularly among those already socially and financially impoverished, and facing other personal challenges (Moffitt 1993; Algood et al. 2011). In residential care settings, this includes inadequately trained, supported, and remunerated staff (Shaw 2012).

The relationship between maltreatment and neuropsychological characteristics common among justice-involved children, including the issue of directionality, is the subject of ongoing investigation. Growing evidence suggests that certain neurodevelopmental and mental disorders associated with increased risk of youth justice contact are oftentimes the result of maltreatment, or at least share with abuse and neglect common etiological pathways (Maclean et al. 2017; Spencer et al. 2005; Sullivan and Knutson 1998). Maternal alcohol consumption during pregnancy is, for instance, implicated in the development of intellectual disability, and at problematic levels is also related to post-birth abuse and neglect (O’Leary et al. 2013). Alterations in affective (emotional) impulse regulation and attention are identified core features of posttraumatic stress disorder (PTSD) and complex PTSD, both of which arise in the context of exposure to traumatic interpersonal victimization (American Psychiatric Association 2013a; Courtois 2004).

Studies that have touched upon this phenomenon have identified a high prevalence of children with neurodisability among populations of “crossover” children who experience contact with both child welfare and youth justice systems (Halemba et al. 2004; Cho et al. 2019; Haight et al. 2016). For instance, Taflan (2017) identified that boys who had been in child welfare care represented 42% of a convenience sample of 720 children in custody across six Irish secure training centers in 2016–17. The study found that these crossover children were more than twice as likely to report that they had a disability compared with other children in youth justice custody (28% vs. 13%) (Taflan 2017). In the US, Halemba et al. (2004) found that 23% of a sample of 204 Arizonan crossover children had a suspected or diagnosed learning disability. Similarly, Haight et al. (2016) noted that between 49% and 74% of four US samples of crossover children (total *N* = 4904) were eligible for Individualized Education Program (special education), though it is unclear what proportion had diagnoses of neurodisability, as opposed to emotional disturbance or other physical/health impairments. Aside from the high prevalence of neurodisability, a prospective longitudinal study of 5002 maltreated Minnesotan youth found that the presence of emotional or behavioral disorders were a significant risk factor for children crossing over into the youth justice system, though it is unclear what range of neurodevelopmental and mental health conditions were captured under this term (Cho et al. 2019).

A few qualitative studies have also drawn attention to the issue of neurodisability among children crossing over between child welfare and youth justice settings. Two exploratory Australian studies have highlighted the high likelihood of maltreated children with cognitive disabilities to be living in residential care, noting the adoption of police responses to behavioral challenges in these settings as a key pathway by which this group comes into justice system contact (Richards and Ellem 2018; Greig et al. 2019). Some researchers have emphasized the influence of type of residential care setting in these situations, with children in generalist residential care more likely to experience police contact than those in specialist disability placements (Richards and Ellem 2018). Though solely focusing on cognitive impairment, which the authors appeared to broadly equate to intellectual disability, a qualitative Australian study of 11 key stakeholders identified key themes underpinning the relationship between cognitive impairment, out-of-home care, and youth justice system involvement (Greig et al. 2019). These included the increased vulnerability of children in care with cognitive impairment to youth justice contact; cognitively impaired childrens’ lack of belonging due to their unique histories and experiences of out-of-home care instability; challenges identifying neurodisability; criminalization of disability-related behaviors; processes steering children to the youth justice system rather than support services; and lack of support for this group, including due to service exclusion (Greig et al. 2019).

Evident from this overview is the dearth of literature examining neurodisability among children at the nexus of child welfare and youth justice systems, despite the identifiable “complex set of vulnerabilities” likely to impact children at these intersections (Dowse et al. 2014, p. 181). Developmental and life course theories anticipate the over-representation of children with neurodisability in child welfare systems, yet the role of child welfare system involvement in mediating the outcomes seen among children with neurodisability in youth justice systems remains unclear.

## The Current Study

The current study aims to expand the evidence base concerning the intersections between childhood adversity, child protection involvement, neurodisability, and youth justice system contact and does so within the Australian context. This article examines differences between crossover children with and without neurodisability in relation to childhood adversity, child protection involvement, and youth offending.

While a greater risk of maltreatment is identified among children with neurodisability in the community (Sullivan and Knutson 2000), there is a dearth of research that examines childhood adversity and protective services involvement among justice-involved children with neurodisability. It is hypothesized that compared to crossover children without neurodisability, those with neurodisability will experience greater cumulative childhood adversity (Hypothesis 1), earlier child protection notifications (Hypothesis 2), more child protection notifications and substantiations (Hypothesis 3), and are more likely to have experienced out-of-home care placement (Hypothesis 4).

Studies have also found earlier youth justice involvement and more violent offending among maltreated youth (Fitton et al. 2018), however it remains unclear if, and to what extent, these outcomes are related to the greater prevalence of neurodisability among maltreated children. It is hypothesized that compared with other crossover children, those with neurodisability will experience earlier police charges (Hypothesis 5), and a greater prevalence of violent offending (Hypothesis 6). In addition to these confirmatory analyses, certain exploratory analyses will be conducted to provide more detailed insight in the following manner. First, given the identification of gender differences (Malvaso et al. 2017a; Ryan et al. 2010), and the prevalence of certain offense types and contexts among maltreated and crossover children (e.g., violent offending and residential care-based offending (Malvaso et al. 2018b; Baidawi and Sheehan 2019a)) exploration of gender differences, and offense types or contexts will be undertaken among the study sample. Second, additional analyses will examine differences in the relationships examined between specific neurodisabilities. Finally, differences in the nature of childhood adversity (i.e., prevalence of specific adverse childhood experiences) will be explored among crossover children presenting with varying neurodisability statuses.

## Methods

This article analyzes data collected as part of the 2016–18 Cross-Over Kids Study conducted in partnership with the Children’s Court in Victoria, Australia. The Victorian Children’s Court comprises a Family Division that hears applications relating to the care and protection of children aged 0–17 years at risk of abuse and neglect, as well as intervention order applications, alongside a Criminal Division that deals with alleged offending of children aged 10–17 years (though children up to 21 years may be subject to youth justice orders).

### Sampling

The sample of crossover children comprised all children (aged 10–17 years at the time of criminal charges) before each of three Victorian children’s criminal courts who had current or historical protective matters in any Victorian Children’s Court. Study courts—including one regional and two metropolitan courts—were purposively selected to form a diverse sampling frame in relation to children’s crime and protective matters, socio-economic status, rurality, and culture. Cases were identified in chronological order of the child appearing before the criminal courts from June 2016 until the quota of 300 cases was filled in April 2017. Excluded were children with non-statutory child protection involvement alone, children solely with interstate child protection involvement, and children presenting to criminal courts only with infringement matters (e.g., failing to wear a bicycle helmet).

### Data Collection

Data were gathered via a manual audit instrument developed by the research team with advice from the Children’s Court. Four data sources were audited for each child: Court-based criminal and family division electronic files, and hard copy criminal and family division files. Criminal division files were those of the criminal matter(s) for which the child was currently before the Court. Family division files were those of the child’s current or most recent protective matter.

### Measures

#### Age

Child’s age at court adjudication of their current criminal matter (*m* = 16.2 years, SD = 1.63, [10–21]).

#### Gender

Child’s gender was coded as 1 = male (68%), 2 = female (31%), 3 = transgender (2%).

#### Child protection involvement

The following data were collected for each child: number of child protection notifications and substantiated notifications (i.e., following protective services investigation); current statutory court order (yes/no); current (yes/no) and historical (yes/no) out-of-home care placement; current (yes/no) and historical (yes/no) out-of-home care placement types including kinship care (extended family carers), foster care (non-family carers) and residential care (non-family carers in group setting). Caregiver relinquishment was coded for children whose caregivers (including by parents, kinship or foster carers) at some point indicated their unwillingness or incapacity to continue to care for the child.

#### Neurodisability

Neurodisability was identified for those children whos case files indicated a diagnosis with any of: intellectual disability, borderline intellectual functioning, learning or communication disorder, attention deficit/hyperactivity disorder, autism spectrum disorder, Tourette syndrome, fetal alcohol spectrum disorder, organic acquired memory disorder, acquired brain injury, or epilepsy.

#### Mental illness

Mental illness was identified for children whose case files indicated a formal diagnosis of mood, conduct, personality, trauma and attachment-related, psychotic, and other mental illnesses.

#### Adverse childhood experiences

Data concerning children’s victimization by direct experiences of physical, emotional, or sexual abuse, or neglect, and exposure to other adverse childhood events were collected from court documents, official child protection reports and other reports presented to the court (e.g., psychological/psychiatric assessments, police reports, pediatric forensic reports). Identification of maltreatment was based on descriptions of abuse and neglect in case files which accorded with World Health Organization and the International Society for Prevention of Child Abuse and Neglect definitions (2006). Although time consuming, this method generated a different picture of children’s maltreatment experiences than would have been gathered by reliance on child protection notification/substantiation data alone. Child protection substantiations were not always observed to be an accurate indicator of child maltreatment, as they indicate legal proof of a *significant risk of harm*, rather than *actual maltreatment* occurring. For instance, *significant risk of physical harm* was often substantiated in relation to adolescent risk-taking behavior (e.g., substantial substance abuse), and therefore was not always indicative of actual *physical abuse*, per se. Exposure to each adverse childhood experience was recorded as binary (yes/no).

##### Parental separation

The child’s biological parents are separated/divorced.

##### Parental death

One or both of the child’s biological parents are deceased.

##### Physical abuse

Intentional use of physical force against the child by a parent or caregiver that resulted in – or has a high likelihood of resulting in – harm to the child’s health, survival, development or dignity. This includes acts such as hitting, beating, kicking, shaking, biting, strangling, scalding or burning a child, with or without the use of weapons.

##### Emotional abuse

Inappropriate verbal or symbolic acts toward the child by a parent or caregiver which fail to ensure a supportive environment, and which have a high probability of damaging the child’s physical or mental health, or the child’s physical, mental, spiritual, moral or social development. This includes patterns of name-calling, belittling, blaming, threatening, frightening, discriminating against or ridiculing; and other non-physical forms of rejection or hostile treatment.

##### Sexual abuse

Involvement of the child in sexual activity that he or she does not comprehend, is unable to give informed consent to, or for which the child is not developmentally prepared, or else that violates the laws or social taboos of society. For the purposes of this analysis, this included direct sexual victimization involving sexual contact with the child, as well as acts of voyeurism, exhibitionism, and exposing the child to adult sexual acts including intercourse and pornography.

##### Neglect

Exposure of a child to isolated incidents, or a pattern of failure over time on the part of a parent or carer to provide for the development and well-being of the child in relation to the child’s health, emotional and physical development, education, supervision, and safety. Examples of neglect included lack of enrollment of children in school, exposing children to hazardous environments (e.g., animal/human urine or fecal matter, or drug paraphernalia), leaving children alone for age-inappropriate periods or with inappropriate adults (e.g., registered sex offenders), not providing food for the child, and not attending to the child’s expressed distress.

##### Family violence exposure

Exposure of the child to the physical, emotional or sexual victimization of another family member. In most cases (87%), family violence was perpetrated by caregivers, including step-parents and kinship carers, but also included acts perpetrated by others including extended family and siblings.

##### Household mental illness

Exposure of the child to a parent or household member with mental illness, identified by formal diagnosis of mood, conduct, personality, trauma and attachment-related, and psychotic disorders.

##### Household substance abuse

Exposure of the child to parent or household member (e.g., step-parents and extended family members) who abused substances (drugs and/or alcohol, including prescription medications).

##### Household criminal justice involvement

Exposure of the child to a parent or household member (including step-parents and siblings) with community or custodial criminal justice system involvement.

##### Adverse childhood experience score

Much like the measurement strategy used in other adverse childhood experience research (Anda et al. 2006), a score was calculated for each child (0–10) based upon their cumulative exposure of each of the above experiences.

#### Offending

Each child’s official police charges (date and offense type) were collected from the court electronic database. Information contextualizing children’s charges and offending were collected from hard copy case files, including qualitative data from police briefs, youth justice reports, and other reports/assessments submitted to the court.

##### Total charges

Each child’s total number of charges (including current and historical matters) was calculated at the date of adjudication (sentencing/dismissal) of the child’s current criminal matter.

##### Violent charges

Violent charges were recorded for children whose charges included offenses against the person classified under Divisions 1–5 of the Australian and New Zealand Standard Offence Classification (ANZSOC) encompassing homicide and related offenses, acts intended to cause injury, sexual assault and related offenses, dangerous or negligent acts endangering persons, and abduction, harassment and other offenses against the person (Australian Bureau of Statistics 2011).

##### Adolescent family violence

Adolescent family violence was recorded for children whose case files outlined their acts of physical and verbal violence, property damage, and threatening or intimidating behavior towards family members, and current or former romantic partners, or where they were charged with contravention of a family violence intervention order.

##### Residential care-based charges

Residential care-based charges were recorded for children whose qualitative case file data (e.g., police briefs, youth justice reports) indicated their acquisition of criminal charges within and in the surrounds of a residential care placement. Charges typically included those related to property damage, assault, making threats, reckless conduct (e.g., throwing an object), and resisting arrest in these settings.

### Data Availability and Quality

Case file audits were fully completed for 91.3% of children. Case file audits were incomplete (13/300 Family Division files and 14/300 Criminal Division files), when the file could not be located or was unavailable because of an ongoing matter at another Court. In these cases, much of the required information was gathered from other reports/files minimizing missing data. Electronic records case file data were consistently available, however information in hard copy files varied depending on the particular assessments and reports provided to the Court. As such, quantitative findings represent minimum prevalence data regarding the variables examined. On the other hand, an advantage of the research approach is that detailed data regarding children’s current and historical circumstances were often obtained from several sources, permitting cross-checking and triangulation for greater reliability.

The mechanism of data missingness was analyzed in relation to neurodisability, that being the variable most central to the study with a notable proportion of missing data. Neurodisability data were available for 93% of the sample (*n* = 279), and there were no differences in age, gender, or number of child protection notifications and substantiations by availability of neurodisability data. While neurodisability data were more likely to be missing from cases based in the regional court, there was no significant difference in the proportion of children with neurodisability at this and the other court locations, suggesting that these data were likely missing at random. Cases with missing neurodisability data were excluded from relevant analyses.

### Data Analysis

Case file data were entered into SPSS24 where bivariate analyses were used to examine differences between crossover children with and without neurodisability (Fisher’s exact test for categorical and *t*-tests for continuous variables, respectively). Logistic regression analyses (controlling for age) were utilized to examine relationships between neurodisability (including specific diagnoses), violent offending, as well as categories of offending that were significantly associated with offending in bivariate associations.

### Sample Characteristics

Sample characteristics, outlined in Table 1, demonstrate that most crossover children were aged between 15 and 17 years, and two-thirds were male. Though ethnicity/race data were collected, they are not reported in the findings due to significant missing data.^1^ Just over one half (57%) of children were under a current child protection order, including 43% who were in out-of-home care, predominantly residential care.

**Table 1:** Sample characteristics

Characteristic	All (*N* = 300)	Males (*n* = 204)	Females (*n* = 94)	*p* (Males vs. Females)
Age (years) m [min, max]	16.2 [10, 20]	16.3 [10, 20]	16.2 [11, 20]	0.629
Current child protection order	57%	57%	57%	1.000
Current out-of-home care (OHC) placement (*n* = 300)				
Yes	43.3%	46.1%	36.2%	0.131
No	43.7%	41.2%	50.0%	0.168
Over 18 years (ineligible for OHC)	13.0%	12.7%	13.8%	0.853
Type of OHC placement (*n* = 130)				
Residential care	69.2%	66.0%	76.5%	0.288
Foster/kinship/permanent care	22.3%	24.5%	17.6%	1.000
Independent living	6.2%	6.4%	5.9%	0.454
Unknown/other	2.3%	3.2%	0%	0.565
Neurodisability (*n* = 279)				
Any neurodisability	48.0%	58.9%	25.3%	0.000
ADD/ADHD	29.0%	37.9%	10.3%	0.000
Learning/communication disorder	22.6%	28.9%	9.2%	0.000
Intellectual disability	17.2%	20.0%	11.5%	0.090
Borderline intellectual functioning	7.2%	10.0%	1.1%	0.006
Autism spectrum disorder	5.7%	8.4%	0.0%	0.004
Other^a^	3.6%	3.7%	3.4%	1.000
Mental illness (*n* = 283)				
Any mental illness	61.2%	59.9%	63.2%	0.691
Mood disorder^b^	28.3%	25.3%	34.5%	0.117
Conduct disorder^c^	20.1%	22.7%	14.9%	0.151
Trauma/attachment-related disorder^d^	19.8%	21.1%	14.9%	0.254
Psychotic disorder^e^	5.3%	5.7%	3.4%	0.561
Personality disorder^f^	3.5%	0.5%	9.2%	0.000
Other mental illness^g^	0.7%	0.5%	1.1%	0.524
Adverse childhood experiences (ACEs) (*n* = 300)				
Any ACE	98.7%	98.0%	100%	0.312
Parental separation	87.7%	87.3%	88.3%	0.852
Household family violence	73.3%	75.0%	70.2%	0.399
Household substance abuse	69.0%	71.1%	63.8%	0.227
Neglect	67.3%	68.6%	64.9%	0.595
Physical abuse	60.3%	59.8%	60.6%	1.000
Emotional/psychological abuse	53.7%	49.5%	61.7%	0.610
Household mental illness	50.7%	51.5%	47.9%	0.619
Household criminal justice system involvement	40.7%	42.6%	37.2%	0.447
Sexual abuse	20.7%	13.2%	36.2%	0.000
Parental death	20.3%	19.6%	22.3%	0.644
Number of ACEs (mean, SD)	5.4, 2.2	5.4, 2.2	5.5, 2.2	0.586

Transgender children (*n* = 2) excluded from male/female columns

*ACE* adverse childhood experience, *ADD* attention deficit disorder, *ADHD* attention deficit hyperactivity disorder, *OHC* Out-of-home care ^a^Tourette syndrome, acquired brain injury, acquired organic memory disorder, fetal alcohol spectrum disorder, and epilepsy

^b^Anxiety, depression, and bipolar disorder

^c^Conduct disorder, behavioral disorder, and oppositional defiant disorder

^d^Post-traumatic stress disorder, complex post-traumatic stress disorder, reactive attachment disorder

^e^Schizophrenia, schizophreniform disorder, psychotic episodes

^f'^Emerging’ borderline personality disorder and antisocial personality disorder

^g^e.g., adjustment disorder

Children’s most recent child protection matters commonly concerned emotional/psychological and/or physical harm (78%), and their current protective risks included ongoing exposure to family violence, mutual family violence between the child and adults, caregiver rejection and abandonment, and concerns related to the child’s welfare while running away from home or care placements, including due to substance abuse, risk of sexual exploitation, and association with peers and adults involved in crime or substance use. Compared with the state-wide population of crossover children (those ever under child protection orders and who were sentenced/diverted in 2016–17), females were slightly under-represented in the study sample (31% vs. 39%), though the proportion who had ever been in out-of-home care was similar across the two studies (79% vs. 81%) (Sentencing Advisory Council 2019).

## Results

Among the sample of crossover children (Table 1), almost one-half (48.0%, *n* = 134) had a recorded neurodisability, most commonly ADD/ADHD (29.0%), learning and communication disorders (22.6%) and intellectual disability (17.2%). Data relating to the severity of intellectual disability was available in 81% of cases (38/47 children), and in most instances (34/38 children or 89%) was classified as mild, while the remaining 11% were classified as moderate. Neurodisability was significantly more prevalent among male compared to female crossover children (58.9% vs. 25.3%, *p* < 0.0001), and crossover children with neurodisability were significantly younger on average (16.0 vs. 16.4 years, *p* = 0.021), though this difference is insubstantial in a practical sense. Consistent with evidence in the general population, comorbidity in neurodisability, and with mental health conditions were each common among crossover children (American Psychiatric Association 2013b). Of crossover children with neurodisability, homotypic comorbidity characterized 55% (*n* = 74) who were each diagnosed with between two and five unique neurodisabilities. Additionally, crossover children with neurodisability were significantly more likely to have a mental health diagnosis compared with crossover children without neurodisability (72.4% vs. 50.3%, *p* = 0.0002), though variability among diagnostic categories was evident. While crossover children with neurodisability were more likely to have been diagnosed with conduct disorder (34.3% vs. 7.6%, *p* < 0.0001), and trauma and attachment-related disorders (posttraumatic stress disorder and reactive attachment disorder) (29.9% vs. 11.0%, *p* < 0.0001), they were less likely to be diagnosed with personality disorder (0.7% vs. 6.2%, *p* = 0.02) compared with other crossover children. No differences were observed in relation to mood disorders or psychotic disorders by neurodisability status.

To help contextualize these empirical findings, it is instructive to consider the case of “Dylan” (name changed for confidentiality), a 15-year old male who came before the youth criminal court with 25 charges ranging from shop thefts to threats to kill, assault and property damage. Many of the current charges were acquired in the context of his residential care placement, entered just prior to Dylan’s 15th birthday. He had one previous criminal matter, comprising 44 charges commencing around the time of this residential care placement, which were similarly primarily related to property damage, threats to kill and assault. Dylan was first notified to protective services at 1 month of age and has been the subject of five notifications and one substantiation relating to risk of physical, emotional/psychological harm, and parental abandonment. He is one of seven siblings, and protective concerns for Dylan related to exposure to family violence, parental mental health concerns, paternal drug and alcohol abuse, and direct physical and emotional abuse of Dylan. Following parental separation, Dylan had little contact with his father, and his mother struggled to care for his emotional and behavioral needs. These needs included “extreme and volatile” behaviors and “angry outbursts” understood to be related to his history of developmental trauma and lack of secure attachment, alongside neurodisability-related processing and regulation challenges. Dylan’s mother subsequently relinquished his care at age seven, at which time he entered a specialist foster care placement and was provisionally diagnosed with autism. Later assessments resulted in Dylan’s diagnosis with ADHD, a mild intellectual disability, speech and literacy disorders. Dylan’s behavioral challenges were noted to escalate particularly in relation to inconsistency in maternal access visits, which were often canceled or delayed by his mother. Dylan struggled to understand why he could not reside with his family, and was described as suffering a sense of rejection and lack of belonging. While the foster care placement was successful for several years during which time Dylan continued to attend specialist schools on a modified program, the carers were unable to respond to Dylan’s escalating needs. The placement broke down just prior to Dylan’s 15th birthday culminating in Dylan’s entry to residential care and receipt of the above police charges.

### Adverse Childhood Experiences and Neurodisability

**Hypothesis 1.** Almost all children (98.7%) had exposure to at least one adverse childhood experience (ACE) noted in their case file. Confirming hypothesis one, crossover children with any neurodisability evidenced higher average ACE scores compared to crossover children without any neurodisability (*m* = 5.9 vs. 5.2, *p* = 0.01) (Table 2). Differences were investigated for the three most prevalent neurodisabilities, which comprised attention deficit disorder/attention deficit hyperactivity disorder (ADD/ADHD), learning and communication disorders, and intellectual disability. There was some variability between neurodisabilities, with crossover children with intellectual disability and learning/communication disorders displaying significantly higher average ACE scores compared to crossover children without these diagnoses, however children with ADD/ADHD did not display higher ACE scores than crossover children without these diagnoses.

**Table 2:** Neurodisability status and average ACE scores among crossover children

	All (*n* = 279)	Males (*n* = 190)	Females (*n* = 87)	Diagnosed males vs. females	Diagnosis vs. no diagnosis	
ACE scores	*m* (SD)	*m* (SD)	*m* (SD)	*t*	*t*	
No neurodisability	5.2 (2.2)	5.1 (2.3)	5.3 (2.3)	0.55	–	
Any neurodisability (*n* = 134)	5.9 (2.1)	5.7 (2.1)	6.7 (1.8)	2.13*	2.48*	(all)
					1.84^†^	(males)
					2.66**	(females)
ADD/ADHD (*n* = 81)	5.8 (2.0)	5.7 (2.1)	6.0 (1.4)	0.39	1.05	(all)
					1.27	(males)
					0.46	(females)
Learning/communication (*n* = 63)	6.2 (1.9)	6.1 (1.9)	7.3 (1.3)	1.69^†^	19.23***	(all)
					2.41*	(males)
					2.15*	(females)
Intellectual disability (*n* = 48)	6.4 (2.0)	6.2 (1.9)	7.3 (1.9)	1.67	3.02**	(all)
					2.21*	(males)
					2.53*	(females)

Transgender children (*n* = 2) were excluded from the above gender analyses. Data in the final column of the table compare crossover children with each neurodisability diagnosis with children without that specific diagnosis, including by gender. Only children with ‘any neurodisability’ are compared with children with ‘no neurodisability’

*ACE* adverse childhood experience

^†^*p* < 0.1; *p < 0.05; **p < 0.01; ***p < 0.001

Several gender differences were identified in the relationships between total ACE scores and neurodisability status for crossover children. First, females with neurodisability had significantly higher ACE scores compared to males with neurodisability (6.7 vs. 5.7, *p* = 0.04). Second, while females with any neurodisability had significantly higher ACE scores than females without neurodisability (*p* = 0.01), the difference in ACE scores for males with and without neurodisability was not quite significant (*p* = 0.07), however this finding obscures differences between specific diagnoses. Males and females diagnosed with each of intellectual disability and learning/communication disorders displayed higher ACE scores than males and females, respectively, without each of these diagnoses. However, similar disparities were not observed among crossover children with and without ADD/ADHD diagnoses.

Examination of individual ACE exposures by neurodisability status indicated that crossover children with any neurodisability were more likely to have been exposed to household substance abuse and mental illness. As shown in Table 3, certain adverse childhood experience exposures were significantly higher among children with certain neurodisabilities. In particular, family violence, household substance abuse, and household mental illness were higher among crossover children with learning/communication disorders and intellectual disability, compared with children without these diagnoses. Crossover children with ADD/ADHD were also significantly more likely to have a household member affected by mental illness, though they were less likely to have experienced parental death. On the other hand, crossover children with any neurodisability were no more likely to have been exposed to any single form of maltreatment (physical, emotional/psychological, and sexual abuse, or neglect) than other crossover children, though these figures do not account for maltreatment severity, recurrence or chronicity.

**Table 3:** Neurodisability (ND) status and ACE exposure among crossover children

ACE exposure (%)	No ND (*n* = 145)	Any ND (*n* = 134)	ADD/ADHD (*n* = 81)	Learning/communication (*n* = 63)	Intellectual disability (*n* = 48)
Any ACE exposure	97.2	100.0	98.0	98.1	100.0
Parental separation	84.8	90.3	88.9	87.3	93.8
Household family violence	69.7	78.4	74.1	84.1*	89.6***
Household substance abuse	63.4	75.4*	72.8	85.7**	83.3*
Household mental illness	43.4	61.2**	61.7*	66.7*	66.7*
Household criminal justice system involvement	39.3	44.0	40.7	49.2	50.0
Neglect	66.9	72.4	70.4	74.6	79.2
Physical abuse	58.6	66.4	69.1	69.8	66.7
Emotional/ psychological abuse	54.5	58.2	64.2	63.5	54.2
Parental death	21.4	19.4	12.3^*^	17.5	27.1
Sexual abuse	20.7	21.6	21.0	22.2	29.2

ACE exposures of children with specific neurodisabilities are compared with those of children without that particular diagnosis, rather than children without any neurodisability diagnosis

*ACE* adverse childhood experience, *ADD* attention deficit disorder, *ADHD* attention deficit hyperactivity disorder, *ND* neurodisability

*p < 0.05; **p < 0.01; ***p < 0.001

### Child Protection Involvement and Neurodisability

**Hypothesis 2.** Confirming hypothesis two, crossover children with neurodisability were, on average, notified to protective services and substantiated in relation to risk of harm more than two years earlier than crossover children without neurodisability (Table 4).

**Table 4:** Neurodisability (ND) status and child protection involvement among crossover children

Child protection involvement	No ND (*n* = 145)		Any ND (*n* = 134)		ADD/ADHD (*n* = 81)		Learning/communication (*n* = 63)		Intellectual disability (*n* = 48)	
	*m*	SD	*m*	SD	*m*	SD	*m*	SD	*m*	SD
Total notifications	7.1	5.6	8.7*	6.8	8.9^†^	7.4	9.1^†^	8.1	10.9***	8.6
Total substantiations	2.2	1.3	2.6*	1.5	2.7	1.5	2.7	1.6	2.7	1.4
First notification (years)	6.8	5.9	4.3**	4.9	4.3*	5.0	4.3*	4.8	2.5***	3.4
First substantiation (years)	9.4	5.9	7.2***	5.7	7.1*	5.5	6.9*	5.5	5.3***	5.1
First OHC placement (years)	11.7	4.7	9.7**	5.1	10.0	5.0	9.8	4.9	8.4**	5.3
	%		%		%		%		%	
Any OHC placement	75.9		84.3^†^		88.9*		88.9*		89.6^†^	
Any residential care placement	41.4		58.2**		64.2**		61.9*		68.8**	
Caregiver relinquishment	33.1		51.5**		59.3***		52.4^†^		47.9	

Characteristics of child protection involvement among children with specific neurodisabilities are compared with those of children without that particular diagnosis, rather than children without any neurodisability diagnosis

*ADD* attention deficit disorder, *ADHD* attention deficit hyperactivity disorder, *ND* neurodisability, *OHC* out-of-home care

^†^*p* < 0.1; *p < 0.05; **p < 0.01; ***p < 0.001

**Hypothesis 3 and 4.** Confirming hypotheses three and four, crossover children with neurodisability also tended to have a greater number of protective notifications (particularly those with intellectual disability), and placement in residential care was more common among crossover children with neurodisability. Crossover children with a specific ADD/ADHD diagnosis were significantly more likely to have experienced relinquishment by caregivers, including parents, kinship caregivers, or foster carers, often resulting in their entry to residential care environments. A greater likelihood of experiencing relinquishment also approached significance for children with learning/communication disorder (*p* = 0.06).

### Offending Among Crossover Children and Neurodisability

**Hypothesis 5.** As hypothesized, a younger onset of offending characterized crossover children with neurodisability compared to those without neurodisability, as well as a greater number of total charges at the time of their court matter (Table 5).

**Table 5:** Age of first charge and total number of charges by neurodisability (ND) status among crossover children

	No ND (*n* = 145)	Any ND (*n* = 134)	ADD/ADHD (*n* = 81)	Learning/communication (*n* = 63)	Intellectual disability (*n* = 48)
Age of first charge (years)	14.7	13.9***	13.7***	13.8*	13.7**
Total charges (mean)	40.1	56.6*	60.7*	61.9*	55.8

*ADD* attention deficit disorder, *ADHD* attention deficit hyperactivity disorder, *ND* neurodisability

*p < 0.05; **p < 0.01; ***p < 0.001

**Hypothesis 6.** In contrast with the study hypothesis, no differences were observed in the prevalence of violent offending (after controlling for age) for children with *any* neurodisability, however crossover children with specific ADD/ADHD diagnoses were 2.6 times more likely have charges related to violent offending compared to other crossover children (Table 6). Further analyses (not reported here) indicated no significant differences by neurodisability status (y/n) in the prevalence of other offense types including any property, drug, justice procedure, public order or driving offenses. On the other hand, crossover children with *any* neurodisability were more than twice as likely to have specific charges of criminal damage and motor vehicle thefts, and to have evidence in their case files of engagement in adolescent family violence, and to have received residential care placement-related charges. Yet, these overall findings obscured disparities between types of neurodisability. For instance, the odds of a child acquiring charges relating to their placement in residential care were significantly higher for children with ADD/ADHD and intellectual disability, but not for those with learning/communication disorder, which only approached statistical significance (*p* = 0.06). Examples of incidents resulting in residential care-based charges included property damage, assault or resisting arrest in these placements.

**Table 6:** Logistic regressions of offending types and neurodisability (ND) status among crossover children (controlling for age)

	Violent charges		Criminal damage charges		Motor vehicle theft		Adolescent family violence		Residential care-based charges	
Neurodisability (ND)	OR	95% CI	OR	95% CI	OR	95% CI	OR	95% CI	OR	95% CI
Any ND (*n* = 134)	1.88	[0.92, 3.83]	2.57**	[1.50, 4.38]	2.21**	[1.32, 3.69]	2.03**	[1.22, 3.38]	2.19*	[1.16, 4.14]
ADD/ ADHD (*n* = 81)	2.59^*^	[1.04, 6.47]	3.24***	[1.69, 6.22]	1.68^†^	[0.97, 2.90]	1.94*	[1.13, 3.32]	2.85**	[1.52, 5.36]
Learning/ Communication (*n* = 63)	2.24	[0.83, 6.02]	1.47	[.84, 2.60]	1.43	[0.80, 2.58]	1.40	[0.78, 2.50]	1.90^†^	[0.97, 3.71]
Intellectual disability (*n* = 48)	1.22	[0.48, 3.12]	2.61*	[1.20, 5.72]	1.89^†^	[0.98, 3.62]	1.54	[0.81, 2.93]	2.32*	[1.13, 4.74]

*ADD* attention deficit disorder, *ADHD* attention deficit hyperactivity disorder, *CI* confidence interval, *ND* neurodisability, *OR* odds ratio

^†^*p* < 0.1; *p < 0.05; **p < 0.01; ***p < 0.001

Further analyses demonstrated that the increased risk of residential care-based charges for children with intellectual disability was not significant after controlling for placement in residential care. Conversely, the increased risk of acquiring residential care-based charges for children with ADD/ADHD diagnoses persisted after controlling for placement in residential care (OR = 2.108, 95% CI [1.029, 4.321], *p* = 0.042). This indicates that crossover children with ADD/ADHD are more likely to acquire residential care-based charges compared with other crossover children placed in these care settings. Conversely, children with learning/communication disorders or intellectual disability faced elevated risk of acquiring these charges due to their greater propensity to be placed in residential care relative to other crossover children.

### Supplemental Analyses

In addition to the above, childrens’ total number of charges by neurodisability status was also examined in a series of negative binomial regressions that accounted for their differing ages. Significant findings were consistent with those of the bivariate analyses presented in Table 5. That is, these regression analyses similarly showed that crossover children with any neurodisability (*B* = 0.396, 95% CI [0.156, 0.636], *p* = 0.001), ADD/ADHD (*B* = 0.416, 95% CI [0.152, 0.679], *p* = 0.002), and learning/communication disorders (*B* = 0.347, 95% CI [0.064, 0.631], *p* = 0.016) each had a significantly greater number of charges after controlling for age.

## Discussion

While studies have described a high prevalence of neurodevelopmental disability among children crossing over between child welfare and youth justice systems (Cho et al. 2019; Haight et al. 2016), this phenomenon has not been explored in any detail. Yet, these are important relationships, particularly in light of the high proportion of children in custodial justice settings who experience neurodisability, childhood maltreatment, and adversity (Hughes 2015; Malvaso et al. 2018a). The current study sought to make an important contribution to a rather small knowledge base, especially within the Australian context, regarding neurodisability among children at the nexus of child welfare and youth justice systems. Several key findings emerged from this investigation, many of which were consistent with the study hypotheses and others that extended these.

First, while definitional variations limit the comparison of neurodisability prevalence between this and other findings, particularly as many relevant studies tend to group together neurodisability with other conditions (e.g., emotional disturbances), the proportion of children with neurodisability in the sample was substantial (48%), compared to that reported by other research examining disability among maltreated children. For instance, two large-scale US studies each reported that 22% of maltreated children had disabilities (Lightfoot et al. 2011; Sullivan and Knutson 2000). As the definitions of disabilities adopted in these studies varied from those of the current study to include emotional, physical and behavioral disabilities, it appears likely that neurodisability is substantially over-represented among crossover children relative to children solely involved with child protection services.

Second, consistent with the study hypothesis, greater cumulative adversity was observed among crossover children with neurodisability relative to other crossover children. It is of interest that children with neurodisability differed from the rest of the crossover cohort not in relation to the prevalence of maltreatment exposure (abuse or neglect), but rather the household adversity they faced. This was particularly apparent in relation to household mental illness, substance abuse, and family violence experienced by crossover children with neurodisability. Official child protection data also illuminated the greater and earlier protective notifications and substantiations made for crossover children with neurodisability. Such findings are consistent with Moffitt’s developmental taxonomy (1993, p. 681) which acknowledges how “many sources of neural maldevelopment co-occur with family disadvantage or deviance”, and extend findings regarding the higher levels of family adversity among children with specific neurodisability diagnoses who are in contact with youth justice systems (Moffitt 1990).

Third, many of the patterns of maltreatment and protective services involvement described among samples of crossover children (see, for instance, Malvaso et al. 2017a, b) are consistent with those described among samples of children with disabilities. These include a greater prevalence of multi-type maltreatment, earlier protective notifications and substantiations, and a greater likelihood of out-of-home care placement among maltreated children with disabilities relative to the broader population of maltreated children (Sullivan and Knutson 2000; Lightfoot et al. 2011). While differences in exposure to any of physical, emotional and sexual abuse and neglect were not apparent between crossover children with and without neurodisability, this is somewhat anticipated since all of the children in the study sample had protective services involvement. The finding of earlier protective notifications, substantiations and care entry for crossover children with neurodisability appears to conflict with other research findings which suggest that later entry into out-of-home care, and entry into care for behavioral reasons, are each risk factors for criminal convictions among child protection-involved youth (Cho et al. 2019; Baskin and Sommers 2011; Cutuli et al. 2016). However, the present findings contrast crossover children with and without neurodisability only; future research should compare the protective services involvement of crossover children with neurodisability, and other maltreated children with neurodisability, to ascertain any differences in their characteristics and pathways. Importantly, the study findings highlight the high levels of parent/caregiver relinquishment experienced by crossover children with neurodisability (particularly ADD/ADHD), their elevated risk of placement in residential care settings, and their greater likelihood of having their behavior criminalized in these environments.

Other important findings related to differences in offending between crossover children with and without neurodisability. A fourth key finding was the earlier onset of offending among crossover children with neurodisability, in accord with previous findings of the importance of neurodisability (and responsiveness to the needs of maltreated children with neurodisability) to the earlier justice system-involvement observed among maltreated and crossover youth (Cho et al. 2019). Fifth, and contrary to the study hypothesis, no difference in the prevalence of any violent offending was observed between crossover children with and without neurodisability. At the same time, children with specific ADD/ADHD diagnoses were more than twice as likely to have been charged with violent offenses relative to other crossover children. Such findings demonstrate the importance of considering specific neurodisability diagnoses in future research at the intersection of child maltreatment, neurodisability and youth offending. Charges relating to criminal damage, motor vehicle thefts, adolescent family violence, and residential care-based offending were all more common among crossover children with neurodisability.

Sixth, the study’s findings also highlight gender differences in the relationships examined. These differences were notable in the prevalence of neurodisability among male and female crossover children, consistent with trends in the general population (American Psychiatric Association 2013b). The significantly lower prevalence of neurodisability observed among females in the current study indicates that neurodisability appears more relevant to understanding the overall link between maltreatment and youth offending among males. Of interest in relation to gender disparities is also the lack of significant difference between the ACE scores of maltreated males with, and without, neurodisability. This finding is unexpected, but again obscures differences between specific diagnoses, which demonstrated that crossover children with learning/communication disorders and intellectual disability (both male and female) had higher ACE scores than male and female crossover children respectively, without these diagnoses. Conversely, children diagnosed with ADD/ADHD did not exhibit significantly higher ACE scores (regardless of gender) compared with children without these diagnoses, contributing to the overall trends observed among males in the sample in this regard. This difference between ADD/ADHD and the other neurodisabilities among crossover children should be examined in future research.

The greater cumulative exposure to adverse childhood experiences among female crossover children with neurodisability also warrants further investigation, and is of interest in the ongoing efforts to disentangle relationships between childhood maltreatment and adversity, neurodisability or other neuropsychological risks, and youth justice system involvement. The study’s results accord with previous findings that indicate males are more vulnerable to exhibiting antisocial behavior in the face of family adversity, particularly that related to inconsistent discipline, household conflict, transience and instability in housing and caregivers, low socio-economic status and single parenting (Moffitt et al. 2001). The findings are also consistent with the thesis underlying recent epigenetic findings that suggest an enhanced vulnerability of males to neurodevelopmental alterations following exposure to prenatal risks such as maternal stress (Nugent and Bale 2018).

While the current study contributes to building upon the nascent research base on neurodisability among children at the nexus of child welfare and youth justice systems, it is not without limitations. First, its reliance on cross-sectional data tempers the conclusions that can be drawn, particularly causative inferences about these relationships. Future research should strive to track these youth into adulthood and follow them up on a variety of life-course outcomes, including antisocial behavior, interpersonal relationships, as well as education and employment outcomes. Secondly, owing to the naturalistic mining of administrative and real world data, the prevalence of neurodisability, mental health and maltreatment variables are likely to be underestimates due to the lack of assessment of many children, including some who were suspected of having a neurodisability. Case file information demonstrated several assessment barriers including a lack of service availability, transience and instability in children’s lives, and some children’s refusal of assessment. Third, given the Australian context, somewhat limited sample size, and missing data it was not possible to examine how other demographic differences played out, including but not limited to Indigenous status, and to a lesser extent race/ethnicity. The latter comparison is especially important in light of research findings from Florida in the US showing that while blacks are more likely than whites to be diagnosed with conduct disorder, they are much less likely to receive necessary treatment (Baglivio et al. 2017). Yet, treatment is critical for helping them overcome their trauma and help to increase the chances of improved adult outcomes. Work in this area is critically important. A final limitation of these findings is their generation in an Australian context, reflecting the particular child welfare and youth justice environments present in the study jurisdiction. Comparable research in international contexts would be of value to ascertaining their generalizability.

The study findings suggest the need for greater child welfare system responsivity to children with a neurodisability and their families. Future research should identify approaches that reduce caregiver relinquishment of this group to residential care (e.g., availability of respite care or other parental supports). Further, while the need for trauma-informed approaches in residential care is well-recognized, the study’s findings suggest that neurodisability-informed approaches will also be essential to reducing children’s criminalization in these settings. The earlier onset of offending among crossover children with neurodisability sees this group being more greatly impacted by jurisdictions’ minimum ages of criminal responsibility, as well as specific legislative provisions relating to justice system involvement younger children and adolescents. In the study jurisdiction, for instance, children who offend under 14 years may be found “*doli incapax”* by the Court, meaning they not held responsible for the offending due to immaturity in their intellectual or moral development (Fitz-Gibbon and O’Brien 2019). Jurisdictions should be aware of, and responsive to the possibility of neurodisability and trauma exposure, and indeed the likelihood of both, among children encountering the youth justice system, particularly at younger ages. Such youth, often by no fault of their own, exercise a significant cost to their families, various social systems, and society more generally (Cohen and Piquero 2009).

## Conclusion

Although neurodisability features significantly across child welfare and youth justice cohorts, little research investigates neurodisability among “crossover” children at the nexus of these systems. The current study found that nearly one-half of crossover children had a neurodisability, and that this group experienced greater cumulative maltreatment and adversity, earlier out-of-home care entry and offending onset, more caregiver relinquishment and residential care placement, and a greater volume of charges. While crossover children with neurodisability were no more likely to have violent charges relative to other crossover children, they had greater odds of being charged with criminal damage and motor vehicle theft, and of being involved with adolescent family violence, and residential care-based offending. The study found that children with neurodisability constitute a vulnerable and over-represented subgroup of children who cross over between child welfare and youth justice systems. Targeted strategies from both child welfare and youth justice systems will likely be necessary to effectively attend to their needs, and may help prevent a continuation of adversity into adolescence, emerging adulthood, and adulthood.

## Footnotes

1 Data were missing for 47% of children (*n* = 141), as these were not recorded consistently in case files. Children for whom data were available were commonly from Aboriginal or Torres Strait Islander (18%, *n* = 55) and Anglo Australian backgrounds (15%, *n* = 44). The proportion of the sample who were of Aboriginal or Torres Strait Islander backgrounds was equivalent to that reported in a state-wide sample of crossover children coming before the Courts in the study jurisdiction (Sentencing Advisory Council 2019).
